# Specific cyclic ADP-ribose phosphohydrolase obtained by mutagenic engineering of Mn^2+^-dependent ADP-ribose/CDP-alcohol diphosphatase

**DOI:** 10.1038/s41598-017-18393-9

**Published:** 2018-01-18

**Authors:** João Meireles Ribeiro, José Canales, Alicia Cabezas, Joaquim Rui Rodrigues, Rosa María Pinto, Iralis López-Villamizar, María Jesús Costas, José Carlos Cameselle

**Affiliations:** 1 Grupo de Enzimología, Departamento de Bioquímica y Biología Molecular y Genética, Facultad de Medicina, Universidad de Extremadura, Badajoz, Spain; 2 Escola Superior de Tecnologia e Gestão, Instituto Politécnico de Leiria, Leiria, Portugal; 3 Present Address: Clínica Docente los Jarales, Av. El Parque c/c Arterial 31, San Diego, 2006 Estado Carabobo Venezuela

## Abstract

Cyclic ADP-ribose (cADPR) is a messenger for Ca^2+^ mobilization. Its turnover is believed to occur by glycohydrolysis to ADP-ribose. However, ADP-ribose/CDP-alcohol diphosphatase (ADPRibase-Mn) acts as cADPR phosphohydrolase with much lower efficiency than on its major substrates. Recently, we showed that mutagenesis of human ADPRibase-Mn at Phe^37^, Leu^196^ and Cys^253^ alters its specificity: the best substrate of the mutant F37A + L196F + C253A is cADPR by a short difference, Cys^253^ mutation being essential for cADPR preference. Its proximity to the ‘northern’ ribose of cADPR in docking models indicates Cys^253^ is a steric constraint for cADPR positioning. Aiming to obtain a specific cADPR phosphohydrolase, new mutations were tested at Asp^250^, Val^252^, Cys^253^ and Thr^279^, all near the ‘northern’ ribose. First, the mutant F37A + L196F + C253G, with a smaller residue 253 (Ala > Gly), showed increased cADPR specificity. Then, the mutant F37A + L196F + V252A + C253G, with another residue made smaller (Val > Ala), displayed the desired specificity, with cADPR *k*_cat_*/K*_M_ ≈20–200-fold larger than for any other substrate. When tested in nucleotide mixtures, cADPR was exhausted while others remained unaltered. We suggest that the specific cADPR phosphohydrolase, by cell or organism transgenesis, or the designed mutations, by genome editing, provide opportunities to study the effect of cADPR depletion on the many systems where it intervenes.

## Introduction

Cyclic ADP-ribose (cADPR) is a messenger that increases cytosolic calcium mainly by ryanodine receptor-mediated release from endoplasmic reticulum and also by extracellular influx through the opening of TRPM2 channels^1–4^. Given the wide variety of cell processes affected by calcium, many physiological and pathological situations can be mediated by cADPR in different systems, including animals, plants and protozoa. In mammals, it is an important player in processes such as: inflammatory and immune responses, including neutrophil chemotaxis^5^ and T cell activation^6^; smooth muscle cell contraction in arteries and bronchi, with participation in the hypoxic pulmonary vasoconstriction^7,8^ and in the pathogenesis of inflammatory/allergic airway diseases^9,10^; myometrium contractility, eventually contributing to delivery^11,12^; myocyte contraction in adult cardiac tissue^13^, participating in angiotensin II- and β-adrenergic-induced cardiac hypertrophy^14,15^ and in isoproterenol-induced arrhythmias^16^; endocrine and exocrine pancreatic secretion^17,18^, although its role in insulin secretion and diabetes is controversial^19^; cell proliferation and differentiation, regulating e.g. expansion of human mesenchymal stem cells and hemopoietic progenitors^20,21^, neuronal differentiation of PC12 cells^22^, and cardiomyocyte differentiation of mouse embryonic stem cells^23^; and social behaviour in mice, including memory formation and spatial learning^24,25^, related to oxytocin secretion^26,27^ and maybe to niacin deficiency^28^. Furthermore, cADPR is involved in egg activation and fertilization in ascidians^29^ and sea urchin^30^, in early development in sea urchin^31^, in abscisic acid signalling in sponges^32^ and plants^33,34^, in cell fission in dinoflagellates^35^, and in *Toxoplasma gondii* pathogenicity^36–38^. This large series of cADPR effects indicates that tools allowing the manipulation of cADPR concentration could be useful in different scenarios.

Concerning cADPR metabolism, in the mollusc *Aplysia* it is synthesized from NAD^+^ by a specific ADP-ribosyl cyclase^39–41^. However, in mammals cADPR is formed by a collateral reaction of NAD glycohydrolases CD38 and Bst-1/CD157 alternative to the direct formation of ADP-ribose. These mammalian proteins catalyse also cADPR turnover to ADP-ribose, by hydrolysis of the *N*^1^-glycosidic bond^42–49^.

**Figure 1:**
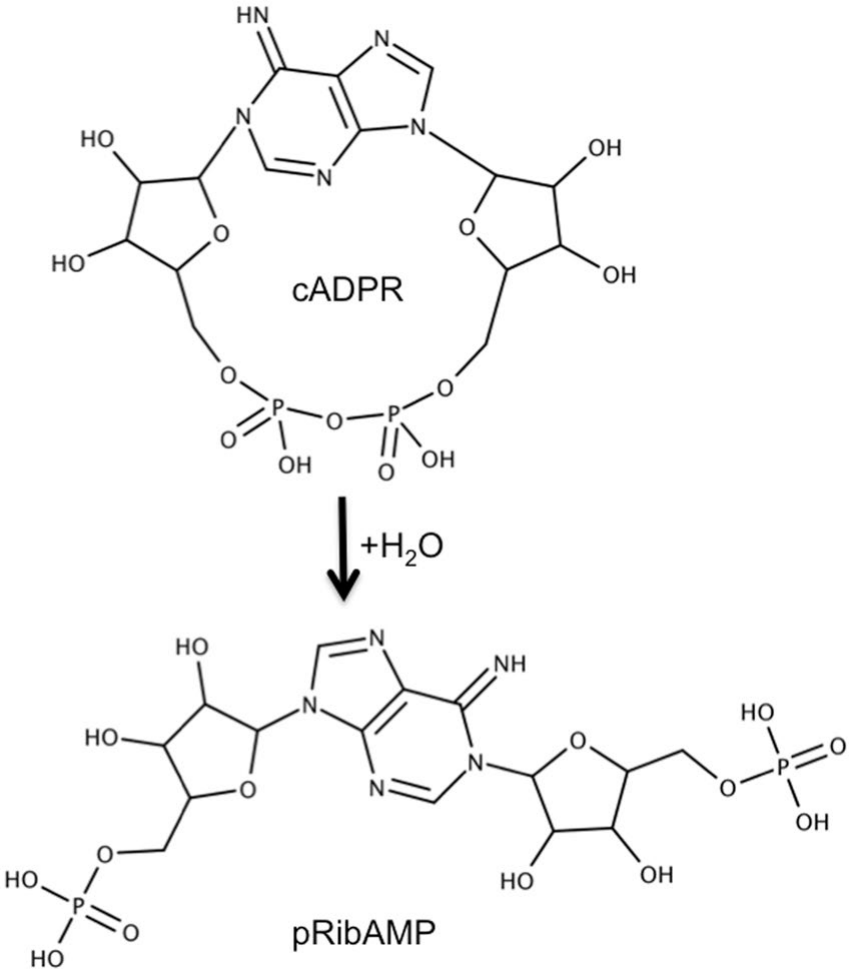
Phosphohydrolysis of cADPR. This is the reaction catalysed by a minor activity of mammalian ADPRibase-Mn^51,55^ and the very major activity of the artificial cADPR phosphohydrolase developed in this study by mutagenic engineering. The left-hand side ribose residue corresponds to the so-called ‘northern’ ribose. MarvinSketch 17.10.0, 2017, ChemAxon (http://www.chemaxon.com) was used for drawing the chemical structures of the reactants.

The phosphoanhydride linkage of cADPR is not attacked by broad-specificity phosphohydrolases^43,50,51^, like snake venom phosphodiesterase, active towards many phosphodiester and phosphoanhydride derivatives of 5′-nucleotides, among them ADP-ribose^52–54^. Therefore, it is noteworthy that mammalian Mn^2+^-dependent ADP-ribose/CDP-alcohol diphosphatases (ADPRibase-Mn; EC 3.6.1.53) hydrolyse the phosphoanhydride linkage of cADPR to *N*^1^-(5-phosphoribosyl)-AMP (pRibAMP) (Fig. 1) with a low but considerable catalytic efficiency (≈150-fold lesser than that of ADP-ribose hydrolysis)^51,55^. This minor activity could be part of a route for the turnover of cADPR, as both this compound and the ADPRibase-Mn product, pRibAMP, have been detected in human red blood cells infected with *Plasmodium falciparum*^56^. The possible physiological role of the enzymatic phosphohydrolysis of cADPR by ADPRibase-Mn is uncertain because the magnitude of the activity is small compared to its major activities. The enzyme has been studied in mammals (*Rattus norvegicus* and *Homo sapiens*)^51,55,57,58^ and in the zebrafish (*Danio rerio*)^59^. It is named after their best substrates (numbers in parenthesis are *k*_cat_*/K*_M_ values, i.e. the specificity constants or catalytic efficiencies): ADP-ribose (3.3–5.9 × 10^5^ M^−1^ s^−1^) and CDP-alcohols (0.1–1.5 × 10^5^ M^−1^ s^−1^), but it hydrolyses also e.g. 2′,3′-cAMP and ADP. In addition, the mammalian ADPRibase-Mn, but not the zebrafish enzyme, hydrolyse cADPR. ADPRibase-Mn specificity is underscored by the lack of activity on ADP-glucose, UDP-glucose, CDP-glucose, CDP, CMP, AMP and 3′,5′-cAMP. Although the efficiency of cADPR phosphohydrolysis by mammalian ADPRibase-Mn (4.0 × 10^3^ M^−1^ s^−1^) is low compared to the phosphohydrolysis of ADP-ribose, it has been earlier pointed out that the ratio between both activities is similar to the ratio of NAD glycohydrolase (i.e. ADP-ribose synthesis) to cADPR synthase activities of CD38 and Bst-1/CD157^51^.

**Figure 2:**
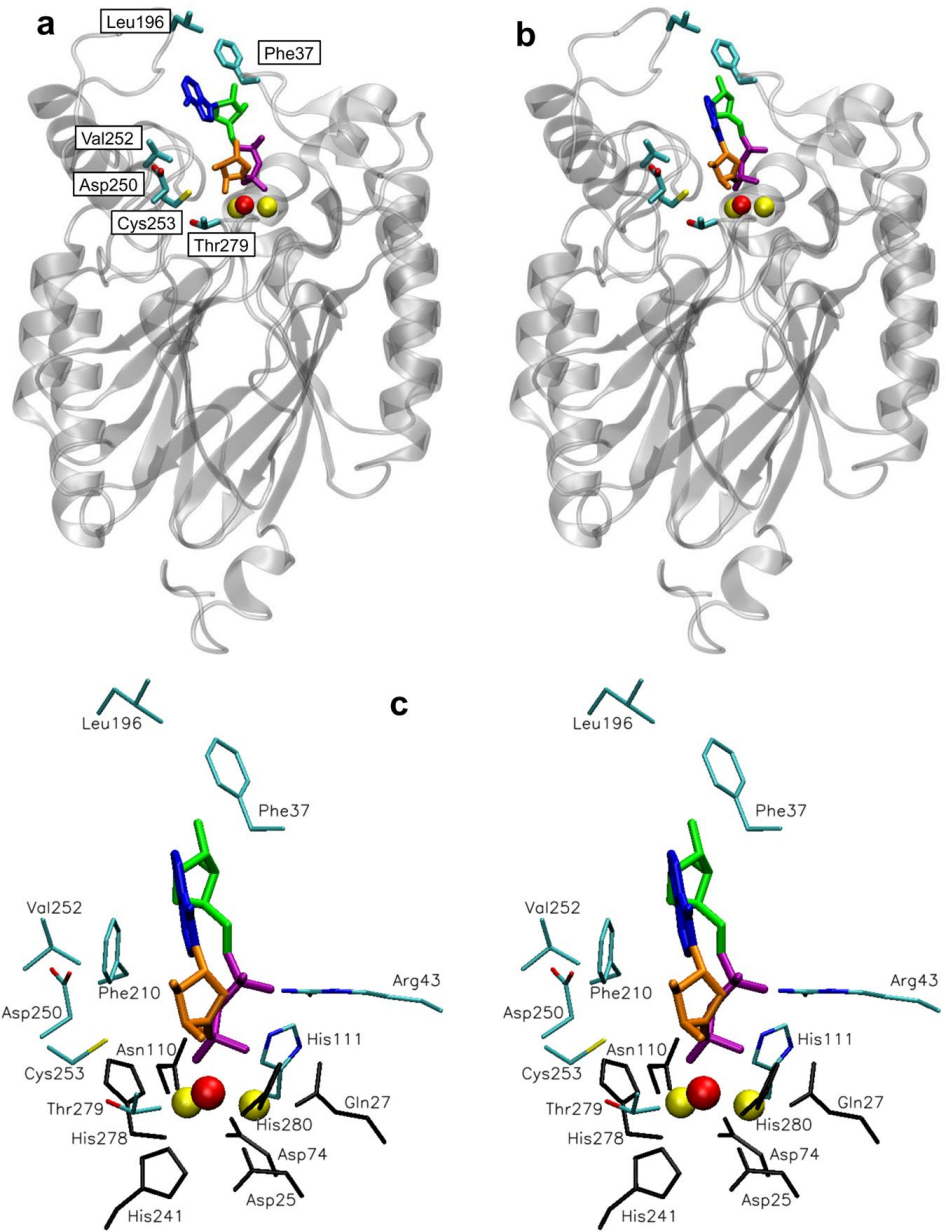
Models of human ADPRibase-Mn with docked substrates. The coordinates for the models were taken from previous work^55^ and the structures were drawn with VMD 1.9.3 (http://www.ks.uiuc.edu/Research/vmd/)^77^ under MacOSX. (**a**,**b**) Full view of ADPRibase-Mn with docked ADP-ribose (**a**) or cADPR (**b**) showing in both cases the amino acid residues that were mutated in this work. (**c**) Stereogram with the detail of cADPR docked to ADPRibase-Mn showing: (i) the ligand (dark blue, adenine; green, ‘southern’ ribose; purple, pyrophosphate; orange, ‘northern’ ribose); (ii) the metals (yellow) of the dinuclear centre with a bridging molecule of water (red) which is assumed to act as nucleophile in the reactions; (iii) amino acid residues bound to the metals (black), of which Gln^27^ and Asp^110^ have been mutated in previous work; (iv) other amino acids mutated previously but not in the current work (Arg^43^, His^111^ and Phe^210^); (v) amino acids mutated in the current work (Phe^37^, Leu^196^, Asp^250^, Val^252^, Cys^253^ and Thr^279^).

ADPRibase-Mn enzymes (Fig. 2) contain the dinuclear metal centre typical of the metallo-dependent phosphatases SCOP2 superfamily^60^, forming within it a family of their own named as ADPRibase-Mn-like (ID 4002589). ADPRibase-Mn proteins constitute also a functional family in the CATH classification, within cluster SC:3 of superfamily 3.60.21.10^61^. The dinuclear centre of ADPRibase-Mn contains a metal-bridging water which, either as such or as hydroxide, it is assumed to be activated by the metals and to attack one of the substrate phosphorus atoms. According to this model, important mechanistic aspects are the correct positioning of the substrate in the active centre for an in-line attack, and transition state stabilization by charge neutralization.

The molecular bases of catalysis and ADP-ribose preference of human ADPRibase-Mn have been investigated by structure-driven mutagenesis based upon substrate docking to a human protein model (Fig. 2)^55^. Two amino acids involved in catalysis have been identified: Arg^43^ and His^111^. The former probably stabilizes the transition state and/or the leaving group, due to the proximity of its positive side chain to the negative phosphoryl group(s) of the substrate. On the other hand, His^111^ seems to be hydrogen bonded to the oxygen of the scissile P-O bond, keeping the substrate in an optimal orientation for in-line attack by the metal-activated nucleophile. Two other residues have been identified as determinants of specificity: Phe^37^ and Cys^253^. The former locates in the rim of the active site entrance, far away from the catalytic core and dimetallic centre. The interaction of the aromatic ring with the nitrogen base of ADP-ribose (Fig. 2a) determines the preference for this substrate. Mutation of Phe^37^ to Ala increases the *K*_M_ for ADP-ribose, with little effect on *k*_cat_ or on the *K*_M_s for other substrates, such that the best F37A-ADPRibase-Mn substrate is CDP-choline instead of ADP-ribose. Cys^253^ is located in the bottom of the substrate cavity, near the catalytic core and dimetallic centre but without interacting with the metals. In models of enzyme-substrate complexes, the thiol group is near the non-nucleosidic ribose of ADP-ribose or the ‘northern’ cADPR ribose. Substitution of Cys^253^ by Ala elicits favourable effects in *k*_cat_ and *K*_M_ for cADPR, thus increasing the specificity constant for this substrate. Interestingly, the combination of F37A and C253A mutations, with a minor contribution also by L196F, results in an ADPRibase-Mn mutant that acts preferentially as cADPR phosphohydrolase, although conserves very substantial activities on the other substrates^55^.

Taking into account the variety of effects mediated by cADPR in multiple biological systems, it was envisaged that an artificial enzyme with specific cADPR phosphohydrolase activity of high catalytic efficiency would be a tool of considerable biotechnological interest. In principle, the behaviour of the triple mutant F37A + L196F + C253A-ADPRibase-Mn^55^ proves the feasibility of altering the substrate specificity of ADPRibase-Mn in favour of its activity towards cADPR and against the other known substrates. Following this thread, additional mutagenic engineering of ADPRibase-Mn was implemented and its effects on substrate specificity and catalytic efficiency were studied, which led to the finding of a quadruple mutant with a very high specificity as cADPR phosphohydrolase.

## Results

### Design of new mutations at or near residue 253 of human ADPRibase-Mn, in the vicinity of the adenine *N*^1^-linked (‘northern’) ribose of cADPR

**Figure 3:**
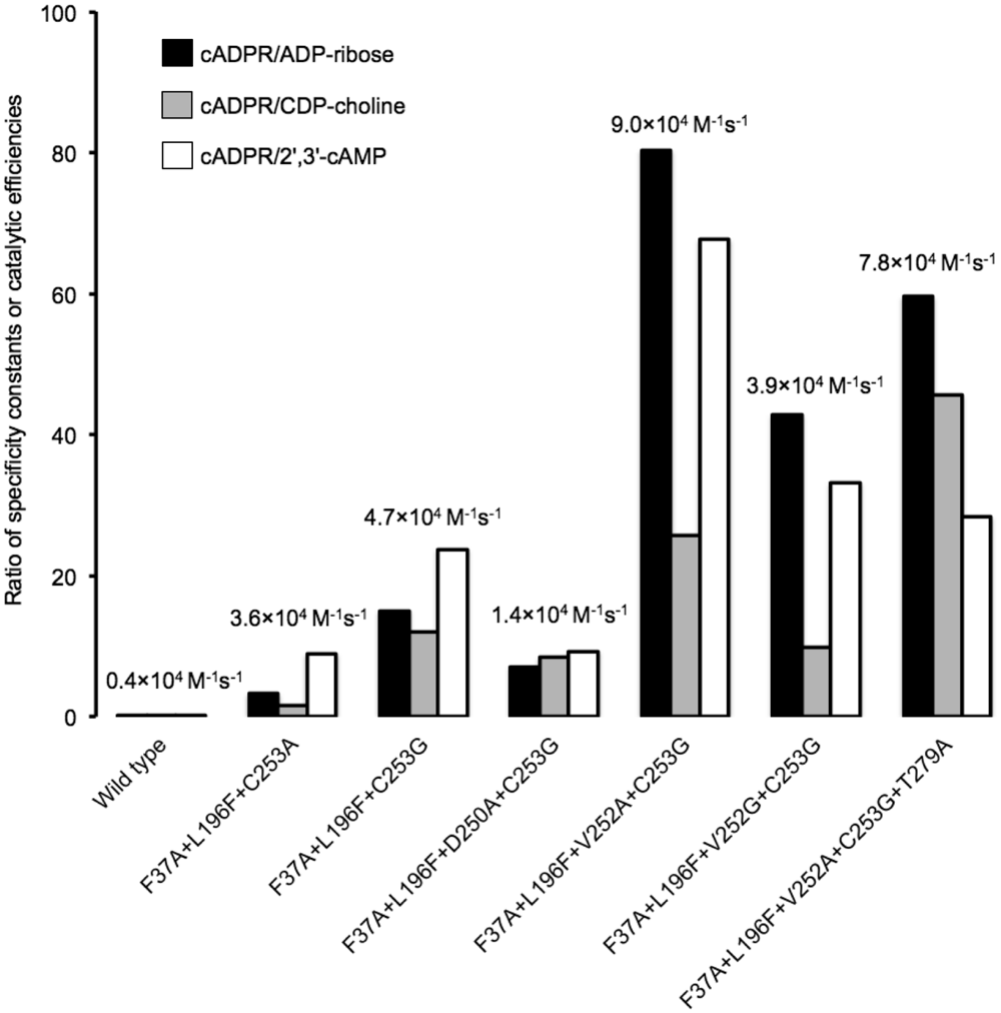
Relative specificity constants or catalytic efficiencies of the constructed mutants of ADPRibase-Mn for cADPR versus other selected substrates. The bars represent the ratios of the *k*_cat_/*K*_M_ values for cADPR versus the *k*_cat_/*K*_M_ values for the other substrates indicated. The numbers above the bars are the absolute *k*_cat_/*K*_M_ values for the hydrolysis of cADPR estimated as described in Supplementary Fig. S1. Data for the wild-type enzyme and for the triple mutant F37A + L196F + C253A were obtained in previous work^55^.

Our recent study of human ADPRibase-Mn^55^ indicates that the specificity of the native enzyme is changed by three mutations (F37A + L196F + C253A) from a clear-cut preference for ADP-ribose, with minor activity on cADPR, towards a relative preference for cADPR. Mutations F37A and L196F diminish the catalytic efficiency with all substrates but less strongly so for cADPR. C253A increases strongly the catalytic efficiency for cADPR but only weakly so for other substrates. In terms of the cADPR/ADP-ribose ratio of efficiencies, this set of combined mutations elicits a 500-fold increase, from 0.007 for native ADPRibase-Mn to 3.2 for the F37A + L196F + C253A mutant. However remarkable this change may be, the resulting preference for cADPR as substrate is not very strong (see below and Fig. 3). The key mutation that favors cADPR phosphohydrolase activity is the removal of the thiol group of Cys^253^ (i.e. C253A). Since in models of cADPR docked to ADPRibase-Mn (Fig. 2) that thiol group is near the northern ribose, the effect of the mutation could be due to the removal of a steric constraint that hinders the positioning of the relatively rigid macrocyclic structure of cADPR in an optimal orientation to undergo attack by the metal-activated nucleophile. Conceivably, this would not occur to the other substrates, which are structurally rather different to cADPR.

To support the role of Cys^253^ as a steric constraint for cADPR, we constructed the single C253V mutant of ADPRibase-Mn, in which cysteine was replaced by the bigger valine residue. This mutant performed worse than the wild-type enzyme as cADPR phosphohydrolase. It hydrolysed ADP-ribose, CDP-choline and 2′,3′-cAMP with higher *k*_cat_ than the wild-type enzyme, while displaying a decreased *k*_cat_ for cADPR (Supplementary Table S1).

Based on this reasoning, novel mutations were implemented in residues near the position where the northern ribose of cADPR docks in the active centre of native ADPRibase-Mn. This included mutating Asp^250^, Val^252^, Cys^253^ and Thr^279^ (Fig. 2b,c). In every case, the initial mutations F37A and L196F were maintained. Of the remaining mutations, C253G was introduced first to construct the triple mutant F37A + L196F + C253G. This change, by putting a side chain smaller than that of alanine in residue 253, was speculatively expected to diminish further the steric constraint to the optimal positioning of cADPR. Since the F37A + L196F + C253G mutant displayed in fact higher efficiency for cADPR than the F37A + L196F + C253A one (see below and Fig. 3), the mutations in Asp^250^, Val^252^ and Thr^279^ were introduced over the F37A + L196F + C253G background. All the additional mutations (D250A, V252A, V252G, T279A) were designed to diminish the size of the affected side chains in search of a combination that, by removing additional steric constraints, enhanced the specificity of the cADPR phosphohydrolase activity. In summary, five new multiple mutants of ADPRibase-Mn were prepared and assayed (Fig. 3).

### Enzymatic characterization of ADPRibase-Mn mutants: a quadruple mutant highly specific as cADPR phosphohydrolase

The five multiple mutants were expressed as recombinant proteins and their *k*_cat_/*K*_M_ values were estimated. Figure 3 shows the ratio of the efficiency recorded with cADPR as the substrate versus the efficiency recorded with other representative substrates (ADP-ribose, CDP-choline and 2′,3′-cAMP). For comparison, the same ratios are also shown for the wild-type enzyme and for the triple F37A + L196F + C253A mutant^55^. For wild-type ADPRibase-Mn, the efficiency ratios are very unfavourable to cADPR, with values of 0.007, 0.027 and 0.16 when the *k*_cat_/*K*_M_ for cADPR is divided by that for ADP-ribose, CDP-choline and 2′,3′-cAMP, respectively. Note that a ratio <1 indicates that cADPR is worse substrate than the other compound. This specificity pattern is already turned upside down in the F37A + L196F + C253A mutant, as the same ratios amount to 3.2, 1.6 and 8.9, making cADPR the best substrate of that ADPRibase-Mn mutant though not by a very large difference^55^.

Concerning the results obtained with the novel mutants, one should first note that changing the methyl group of Ala^253^ to the smaller side chain of Gly^253^ afforded considerable increases of those efficiency ratios in favour of cADPR: 15.0, 12.0 and 23.7, respectively. These changes were accounted for by a slight 1.3-fold increase of the efficiency with cADPR and a 2–6-fold decrease of the efficiency with the other substrates (Table 1).

**Table 1:** Substrate specificity of the quadruple ADPRibase-Mn mutant F37A + L196F + V252A + C253G supporting its denomination and use as specific cADPR phosphohydrolase. The specificity constants (or catalytic efficiencies, *k*_cat_/*K*_m_) are means ± S.D. of three experiments.

Mutations to ADPRibase-Mn	F37A + L196F + C253A		F37A + L196F + C253G		F37A + L196F + V252A + C253G (“specific cADPR phosphohydrolase”)			
	Specificity constant		Specificity constant		Specificity constant		Ratio of specificity constants (cADPR/alternative substrate)	
Substrate	(*M*^*−1*^ *s*^*−1*^)	Fold change	(*M*^*−1*^ *s*^*−1*^)	Fold change	(*M*^*−1*^ *s*^*−1*^)	Fold change	Ratio	Fold change
cADPR	35500 ± 4300	↑9	46500 ± 8400	↑12	90000 ± 7400	↑23	—	—
ADP-ribose	11000 ± 1700	↓50	3100 ± 350	↓180	1120 ± 75	↓490	80	↑11270
CDP-choline	22000 ± 2600	↓7	3870 ± 420	↓40	3500 ± 170	↓44	26	↑1012
CDP-ethanolamine	5800 ± 700	↓8	1450 ± 85	↓31	1740 ± 180	↓26	52	↑598
CDP-glycerol	22000 ± 4000	↓3	6170 ± 560	↓10	3870 ± 80	↓17	23	↑391
2′,3′-cAMP	4000 ± 800	↓6	1960 ± 80	↓12	1330 ± 70	↓18	68	↑414
Ap2A	Not assayed	—	800 ± 65	↓43	420 ± 35	↓83	214	↑1909
NAD^+^	Not assayed	—	1100 ± 6	↓18	630 ± 40	↓31	143	↑713
NADH	Not assayed	—	700 ± 25	↓30	470 ± 60	↓45	191	↑1035
FAD	Not assayed	—	4600 ± 750	↓120	1620 ± 180	↓341	56	↑7843
NADP^+^	Not assayed	—	Not assayed	—	<50	↓>7	>1800	↑>158
NAADP	Not assayed	—	Not assayed	—	<50	↓>3	>1800	↑>67
ATP	Not assayed	—	<200	—	<200	—	>450	—
ADP	<200	—	<200	—	<200	—	>450	—

Data for the triple mutants F37A + L196F + C253A^55^ and F37A + L196F + C253G (this work) are shown for comparison. Changes (↑, increase; ↓, decrease) of the absolute specificity constants or of the ratio of specificity constants of cADPR relative to alternative substrates, are expressed versus the values for the human wild-type ADPRibase-Mn either reported previously^55^ or obtained in this work when not available.

Taking the behaviour of the triple mutant F37A + L196F + C253G as the baseline, one should note the opposing effects of additional mutations in Asp^250^ and Val^252^. The quadruple mutant F37A + L196F + D250A + C253G showed a detrimental effect of the D250A substitution on the efficiency with all substrates (1.3–3.4-fold decrease), and more markedly so for cADPR, such that the substrate efficiency ratios were less favourable than for the triple mutant F37A + L196F + C253G. In contrast, the quadruple mutant F37A + L196F + V252A + C253G showed detrimental effects of the V252A substitution on the efficiency with ADP-ribose, CDP-choline and 2′,3′-cAMP (1.1–2.8-fold decrease) while it increased 2-fold the efficiency with cADPR. The remarkable result of this mutation set was that F37A + L196F + V252A + C253G-ADPRibase-Mn displayed substrate efficiency ratios highly favourable to cADPR: 80, 26 and 68, respectively when compared with ADP-ribose, CDP-choline and 2′,3′-cAMP (Fig. 3).

Further mutations were tested but they did not improve these figures. In contrast to what was observed with alanine and glycine substitutions at Cys^253^, the substitution of Val^252^ by glycine (F37A + L196F + V252G + C253G mutant) did not provide further increase of the specificity for cADPR compared to the substitution of Val^252^ by alanine. Finally, an additional mutation was introduced at Thr^279^. The resulting quintuple mutant F37A + L196F + V252A + C253G + T279A showed an increased efficiency ratio cADPR/CDP-choline relative to its progenitor F37A + L196F + V252A + C253G. However, it was at the cost of a loss of absolute catalytic efficiency on cADPR and of the efficiency ratios cADPR/ADP-ribose and cADPR/2′,3′-cAMP.

Taking into account all these results, the quadruple mutant F37A + L196F + V252A + C253G was chosen for detailed characterization with a series of known substrates of ADPRibase-Mn^55,57,58^ (see the Supplementary Fig. S1) and other compounds of interest. The resulting specificity is described in Table 1 in terms of absolute and relative values of *k*_cat_/*K*_M_. The engineered enzyme hydrolysed cADPR 23–26-fold more efficiently than CDP-glycerol and CDP-choline, which ranked second and third to cADPR, respectively, with even higher preference with respect to the other substrates. NAADP (another calcium regulator formed also by the cADPR synthase and CD38^4,62^), its precursor NADP^+^, ATP and ADP were tested as substrates with negative results. For comparison, Table 1 shows also data for the previously described mutant F37A + L196F + C253A^55^, and for F37A + L196F + C253G, which served as an intermediate step towards the highly specific cADPR phosphohydrolase.

### Demonstration of cADPR phosphohydrolase specificity in complex substrate mixtures

**Figure 4:**
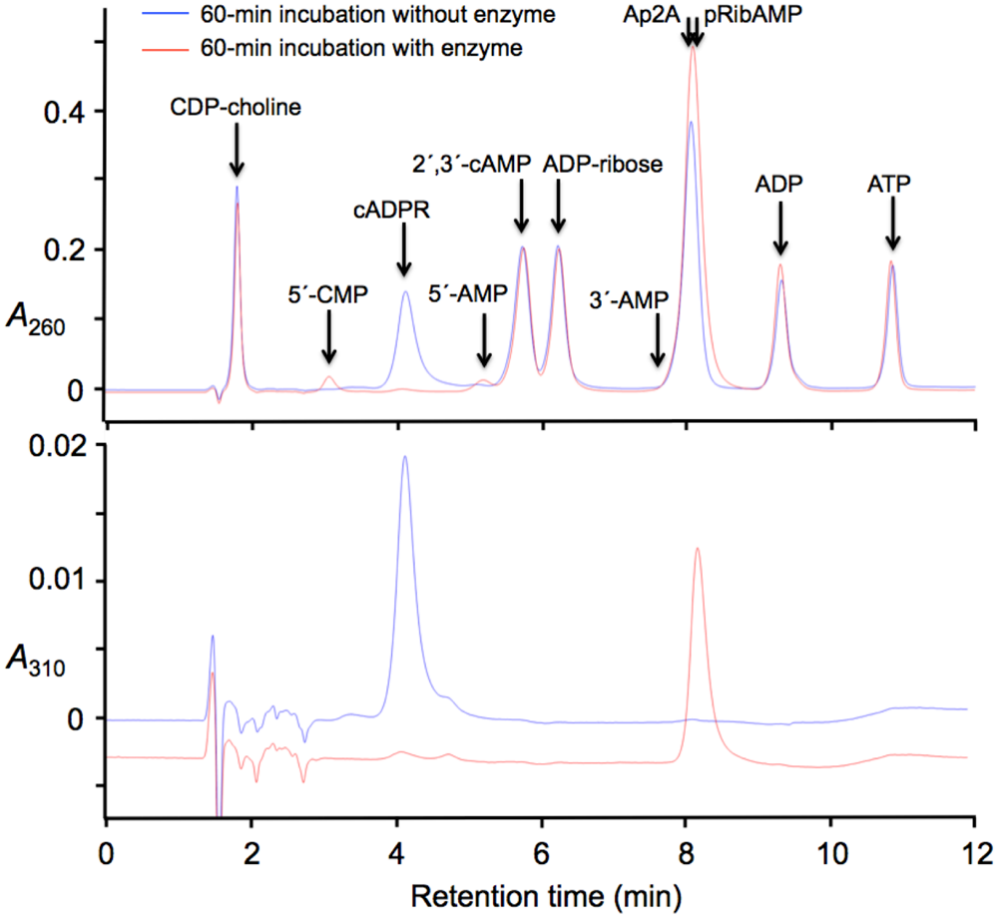
Demonstration of the specificity of cADPR phosphohydrolase in complex nucleotidic mixtures. Mixtures of CDP-choline, cADPR, 2′,3′-cAMP, ADP-ribose, Ap2A, ADP and ATP, each at 0.1 mM concentration were incubated for 60 min at 37 °C in the absence or in the presence of 1 µg ml^−1^ of the specific cADPR phosphohydrolase under otherwise standard conditions. The chromatograms were run with buffers at pH 8.5 as described in Methods, and monitored at 260 nm and 310 nm. The retention times of all the substrates used and of most potential products expected (5′-CMP from CDP-choline; 5′-AMP from ADP-ribose, Ap2A and ADP; 3′-AMP from 2′,3′-cAMP) were determined with samples of the standard compounds chromatographed individually. The pRibAMP produced by phosphohydrolysis of cADPR was characterised in previous work^51^.

To test the specific action on cADPR even in the presence of potentially competing substrates, the engineered enzyme was incubated with mixtures of nucleotides. Figure 4 shows the HPLC analysis of a mixture containing 0.1 mM each of cADPR, ADP-ribose, CDP-choline, 2′,3′-cAMP, Ap2A, ADP and ATP incubated with or without enzyme. All the nucleotidic compounds included in the mixture and their potential phosphohydrolytic products, at least the moieties that contain the nitrogen base, absorb at 260 nm, whereas only cADPR and its phosphohydrolytic product pRibAMP does so at 310 nm. Under the chromatographic conditions chosen, the no-enzyme control displayed seven well-resolved peaks of *A*_260_, each identified with one of the seven compounds included in the mixture, while in the trace recorded at 310 nm only one significant peak appeared, that corresponded with cADPR. In the incubation run in the presence of the artificial cADPR phosphohydrolase, the only compound clearly consumed was cADPR, which was quantitatively converted to pRibAMP (in the trace recorded at 260 nm, this product nearly but not exactly coincided with Ap2A). In agreement with the expected specificity of cADPR phosphohydrolase, the other six compounds remained almost unaltered. Only minor amounts of 5′-CMP and 5′-AMP were detected as products. This could be accounted for by a 5% hydrolysis of CDP-choline, and by a summed-up 1% hydrolysis of ADP-ribose and Ap2A. HPLC analyses of single nucleotides incubated with cADPR phosphohydrolase are shown in Supplementary Fig. S2 to confirm the nature of the products expected in less complex reaction mixtures. These experiments demonstrate clearly the ability of cADPR phosphohydrolase to act specifically in the presence of other nucleotidic compounds.

## Discussion

Emphasis must be put on the overwhelming effect of the F37A + L196F + V252A + C253G mutation set on the specificity of ADPRibase-Mn: compared to the wild-type enzyme, the substrate specificity or efficiency ratio cADPR/ADP-ribose increased >10^4^-fold, from a ratio of 0.007 to as high as 80. For the rest of substrates, the results indicated efficiency ratios of ≈20 to >1800 favourable to cADPR, representing increases of ≈400–10000-fold with respect to the wild-type ADPRibase-Mn (Table 1). This picture portrays the conversion of a native enzyme defined as ADP-ribose/CDP-alcohol diphosphatase to an engineered enzyme that features as specific cADPR phosphohydrolase. This conversion is remarkable not only in terms of relative specificity or efficiency, when cADPR is compared to the other substrates, but also in a >20-fold increase of the absolute catalytic efficiency for the phosphohydrolysis of cADPR, from 4 × 10^3^ M^−1^ s^−1^ of wild-type ADPRibase-Mn to near 10^5^ M^−1^ s^−1^ of the engineered enzyme. One should recall that the median of the distribution of catalytic efficiencies in the enzymosphere lies at ≈10^5^ M^−1^ s^−1^, and 60% of the enzymes with known catalytic efficiencies fall in the 10^4^–10^6^ M^−1^ s^−1^ range^63^. Therefore, the artificial cADPR phosphohydrolase seems well fit to act on cADPR *in vitro* and *in vivo*. The action *in vitro* was made manifest in experiments performed with mixtures of nucleotides, when the artificial enzyme was able to exhaust cADPR almost without affecting the other nucleotides (Fig. 4).

The change of specificity from wild-type ADPRibase-Mn to the specific cADPR phosphohydrolase is caused by mutations in amino acids apparently involved in substrate binding. In fact, considering those substrates for which separate *k*_cat_ and *K*_M_ values were obtained (cADPR, ADP-ribose, CDP-choline, 2′,3′-cAMP; see Supplementary Fig. S1), it can be appreciated that the very high specificity for cADPR of the engineered enzyme depends on a relatively low *K*_M_ value (≈140 µM for cADPR, and ≈12400 µM, 10600 µM and 8700 µM for the others) with little differences among *k*_cat_ values (≈13 s^−1^ for cADPR, and ≈14 s^−1^, 37 s^−1^ and 12 s^−1^ for the others). None of the four mutated amino acids in the artificial specific cADPR phosphohydrolase (Phe^37^, Leu^196^, Val^252^, Cys^253^) appears to be directly involved in enzyme mechanism nor is part of the dinuclear centre. Actually, from earlier work^55^ it follows that mutations of amino acids involved in mechanism (Arg^43^, His^111^) or in the dinuclear metallic centre (Gln^27^, Asn^110^) do not provide any increase of specificity for cADPR.

The artificial specific cADPR phosphohydrolase constructed in this work makes a potentially useful tool for *in vitro* analytical applications, but overall it may be useful for *in vivo* functional studies of cADPR. This could be implemented by cell or organism transgenesis with cADPR phosphohydrolase cDNA under the control of an appropriate promoter, or by editing the ADPRibase-Mn gene (ADPRM) to introduce the F37A, L196F, V252A and C253G mutations (Phe^37^ and Leu^196^ in the ADPRM exon 2; Val^252^ and Cys^253^ in exon 4). The effects of cADPR removal are currently investigated by ablation of the CD38 gene^64,65^, but this does not always produce clean results. In some tissues of CD38 knockout mice, cADPR is far from being fully depleted^5,14,65–70^, most likely because other enzymes can also produce it^46,69,71,72^. The specific cADPR phosphohydrolase, if active *in vivo*, should deplete cADPR independently of its enzymatic source. This would probably occur with little effect on the other CD38 reactants (NAD^+^, ADP-ribose and NAADP) at variance of what happens with the CD38 knockout^14,16,67,68,73^. Anyhow, the use of cADPR phosphohydrolase, even if successfully removing cADPR, will not be without pitfalls. Particularly, one should consider that cADPR would be converted to pRibAMP, which is a product of unknown fate and effects in animal cells. Concerning the minor activities of cADPR phosphohydrolase on substrates other than cADPR, it must be remarked that they would not probably represent novel intracellular activities, since they are displayed by wild-type ADPRibase-Mn with much higher efficiencies (Table 1). Therefore, in transgenesis experiments those activities would bring about minor increases of background levels (unless the recombinant protein were heavily overexpressed), whereas in the case of ADPRM gene editing, they could be lower than in the non-edited cells. All in all, we believe that the *in vivo* expression of cADPR phosphohydrolase activity can provide excellent opportunities to study the effect of cADPR depletion as an alternative or as a complement to CD38 ablation.

## Methods

### Plasmids, enzymes and substrates

Plasmids pGEX-6P-3-hADPRM, harbouring the cloned human liver cDNA of the wild-type ADPRibase-Mn, and pGEX-6P-3-F37A + L196F-hADPRM, a double point mutant used as template for mutagenesis, were obtained as described^55^. Other plasmids were obtained by mutagenesis as described below. PfuTurbo DNA Polymerase from Agilent was used to generate mutated chains, and DpnI from New England Biolabs was used to degrade the methylated templates. The PreScission^TM^ protease was from GE Healthcare purchased through the VWR vendor in Spain. Phosphatase alkaline grade I used as auxiliary enzyme was a former Roche preparation purified from calf intestine purchased from Sigma-Aldrich. Substrates used to study the artificial cADPR phosphohydrolase and other ADPRibase-Mn mutants were obtained, including cADPR purified from the commercial preparation, from the sources described elsewhere^51,58,59^, except diadenosine diphosphate (Ap2A, Sigma), nicotinic acid-adenine dinucleoside phosphate (NAADP, Sigma), and NADP^+^ (Roche, formerly Boehringer).

### Mutagenesis

Mutations of coding sequences were implemented by the *QuikChange* protocol (Stratagene) using ad-hoc mutagenic primers and plasmid templates described in Table 2. The correctness of the full coding sequences obtained by mutagenesis was confirmed by double-strand sequencing in the Servicio de Genómica, Instituto de Investigaciones Biomédicas Alberto Sols, Consejo Superior de Investigaciones Científicas-Universidad Autónoma de Madrid, Madrid.

**Table 2:** Primers and plasmid templates used to construct ADPRibase-Mn mutants

	Mutations	Forward mutagenic primers^a^	Template
1	F37A + L196F + C253G	CCGGACGCGTCTGACAATGTGGGCCTGGCCTGGAACTACAG	F37A + L196F-wt^b^
2	F37A + L196F + D250A + C253G	CCCATTTACCCGGACGCGTCTGCAAATGTGGGCCTGGCCTGGAAC	1
3	F37A + L196F + V252A + C253G	CCCGGACGCGTCTGACAATGCTGGCCTGGCCTGGAACTACAGAG	1
4	F37A + L196F + V252G + C253G	CCCGGACGCGTCTGACAATGGTGGCCTGGCCTGGAACTACAGAG	1
5	F37A + L196F + V252A + C253G + T279A	GGATCCTCAGAGTAGCCACCATCATGTGCGTGACCAGC	3
6	C253V	CGGACGCCTCTGACAATGTGGTCCTGGCTTGGAACTACAG	Wild-type^b^

^a^Reverse mutagenic primers were the exact reverse complement of those shown in the table

^b^Template described elsewhere^55^.

### Protein expression and purification

Human recombinant ADPRibase-Mn and its mutants (all with the N-terminal extension GPLGSPNSRVD) were expressed, from pGEX-6P-3-hADPRM or from mutant plasmids, in *E*. *coli* BL21 cells, followed by affinity purification on GSH-Sepharose and specific proteolysis of the GST fusion protein with the PreScission protease to remove the GST tag^55^. Protein content was assayed in the final preparations^74^ and purity was estimated by image analysis of Coomassie Blue-stained SDS-PAGE gels with the GelAnalyzer 2010 software (http://www.gelanalyzer.com). In every case, a single major band of the expected size (i.e. ≈40.5 kDa) accounted for 64%–82% of protein in the gel lane. Considering that wild-type ADPRibase-Mn is 70% pure, the purity ratios of the mutants versus the wild-type ranged 0.91–1.17. Therefore, the minor purity differences among the mutants and with respect to the wild-type do not affect to an important extent the estimation of the *k*_cat_/*K*_M_ changes caused by the mutations.

### Enzyme assays

All the activities assayed correspond to phosphohydrolytic reactions of phosphoanhydride linkages except for the phosphodiesterase activity on 2′,3′-cAMP. The hydrolysis of cADPR, NADP^+^ and NAADP were assayed by HPLC measuring respectively the formation of pRibAMP, nicotinamide mononucleotide (NMN) or nicotinic acid mononucleotide (NAMN) as products (see below). The hydrolysis of the other substrates was assayed by colorimetric estimation of the amount of inorganic phosphate^75^ liberated from reaction products in the presence of an excess of alkaline phosphatase in the reaction mixture, except that for measuring the activities on ADP and ATP alkaline phosphatase was omitted.

The reaction mixtures contained, in a final volume of 0.1 ml, 50 mM Tris-HCl, pH 7.5 at 37 °C, 0.4 mM MnCl_2_, 6.5 units ml^−1^ of alkaline phosphatase (only when needed, see above), 0.1 mg ml^−1^ bovine serum albumin, and variable amounts of the enzyme sample to be assayed. The reactions were initiated by addition of substrate at the required concentration after a 5-min preincubation of the reaction mixture at 37 °C. Enzyme incubations were terminated either by addition of a phosphate reagent^75^ or by injection in a HPLC column (see below). All the assays were run at 37 °C, under conditions of linearity with respect to incubation time and enzyme amount. Controls without enzyme and/or substrate were run in parallel to full reaction mixtures.

### Estimation of specificity constants or catalytic efficiencies (*k*_cat_/*K*_M_)

Two different methods were used to estimate the specificity constants or catalytic efficiencies on different substrates (see Supplementary Fig. S1). On the one hand, reaction kinetics was often profiled by measuring initial rates at serial substrate concentrations that approached at least a substantial fraction of half saturation. This allowed to estimate *k*_cat_ and *K*_M_ separately by nonlinear regression adjustment of the Michaelis-Menten equation to the data points, and thus to calculate *k*_cat_/*K*_M_. On the other hand, in a simpler approach, *k*_cat_/*K*_M_ was estimated from reaction rates obtained well below the *K*_M_, when rate is (near) proportional to substrate concentration. Reaction rates were determined with different enzyme amounts at two substrate concentrations chosen to obtain a near-linear response versus substrate concentration. This does not provide separate estimations of *k*_cat_ and *K*_M_, but under these conditions the Michaelis-Menten slope equals *k*_cat_/*K*_M_^76^. The two methods used to estimate *k*_cat_/*K*_M_ gave similar results whenever both were applied to the same enzyme and substrate.

### High performance liquid chromatography

Ion-pair reverse-phase HPLC was used to analyse the phosphohydrolysis of cADPR to pRibAMP, NADP^+^ to NMN, and NAADP to NAMN, with a HP-1100 chromatograph (Agilent) equipped with a manual injector, a 20-µl injection loop and a diode array detector. Chromatogram analyses were performed with the HP ChemStation software.

The assay of the phosphohydrolysis of cADPR was conducted by a modification of the method previously described^51,55^. The analyses were performed at 0.5 ml min^−1^ in a 200 mm × 2.1 mm octadecylsilica column (Hypersil ODS; Agilent) with a 20 mm × 2.1 mm guard column of the same material. For reaction mixtures aimed to assay the kinetics of cADPR phosphohydrolase, after sample injection, the chromatographic runs were developed with a 10-min linear gradient of 5 mM–52.5 mM phosphate, using sodium phosphate buffers adjusted at pH 8.5, and containing 20 mM tetrabutylammonium bromide and 10% (vol/vol) methanol. For reaction mixtures aimed to demonstrate the phosphohydrolysis of cADPR in the presence of other nucleotidic compounds, the chromatographic runs were developed with a 2-min isocratic elution in 5 mM phosphate, followed by a 4-min linear gradient up to 43 mM phosphate, a 2-min linear gradient up to 100 mM phosphate, and finished with a 4-min isocratic wash, using sodium phosphate buffers like above. The elution profiles were monitored at 260 nm and 310 nm, the latter allowing the detection of compounds containing *N*^1^-ribosyl-adenine, like cADPR and its phosphohydrolytic product pRibAMP. Reaction rates were estimated from the cADPR-to-pRibAMP percent conversion.

For the assay of the phosphohydrolysis of NADP^+^ and NAADP, the analyses were run at 1 ml min^−1^ in a 150 mm × 4 mm octadecylsilica column (Kromasil 100; Teknokroma, Sant Cugat del Vallés, Barcelona, Spain) with a 10 mm × 4 mm guard column of the same material. The chromatographic runs were developed with a 5-min linear gradient of 5 mM–100 mM phosphate, followed by a 3-min isocratic wash, using sodium phosphate buffers (pH 7.0) with 5 mM tetrabutylammonium bromide and 20% (vol/vol) methanol. The elution profiles were monitored at 260 nm. Reaction rates were estimated either from the accumulation of NMN (retention time, t_R_, 2.0 min) by hydrolysis of NADP^+^ (t_R_, 4.2 min) or from the accumulation of NAMN (t_R_, 3.1 min) by hydrolysis of NAADP (t_R_, 5.2 min). The amounts of NMN or NAMN formed were estimated by comparison to known quantities of the standard products.

### Data and materials availability

The DNA sequence coding for the quadruple mutant (F37A + L196F + V252A + C253G) of human ADPRibase-Mn with the N-terminal extension GPLGSPNSRVD, here characterised as artificial specific cADPR phosphohydrolase, has been deposited in GenBank with accession number MF447900. The rest of data generated or analysed during this study are included in this published article and its Supplementary Information files, or are available from the corresponding author. The plasmid pGEX-6P-3-F37A + L196F + V252A + C253G-hADPRM, encoding the artificial cADPR phosphohydrolase, and the rest of materials generated during this study are available from the corresponding author upon reasonable request.

## Electronic supplementary material

Supplementary Table and Figures
