# Supplementary material for: Specific cyclic ADP-ribose phosphohydrolase obtained by mutagenic engineering of Mn2+-dependent ADP-ribose/CDP-alcohol diphosphatase
Source: Sci Rep. 2018 Jan 18;8:1036. doi: 10.1038/s41598-017-18393-9 (PMC5773619; doi:10.1038/s41598-017-18393-9)
Supplement: Supplementary file 1 — Supplementary Table and Figures [file 41598_2017_18393_MOESM1_ESM.pdf]

SUPPLEMENTARY MATERIAL

Content

Supplementary Table S1

Supplementary Figure S1

Supplementary Figure S2

Specific cyclic ADP-ribose phosphohydrolase obtained by mutagenic engineering of Mn^{2+}-dependent ADP-ribose/CDP-alcohol diphosphatase

João Meireles Ribeiro,[1] José Canales,[1] Alicia Cabezas,[1] Joaquim Rui Rodrigues,[2] Rosa María Pinto,[1] Iralis López-Villamizar,[1,3] María Jesús Costas,[1] José Carlos Cameselle[1]*

[1]Grupo de Enzimología, Departamento de Bioquímica y Biología Molecular y Genética, Facultad de Medicina, Universidad de Extremadura, Badajoz, Spain
[2]Escola Superior de Tecnologia e Gestão, Instituto Politécnico de Leiria, Leiria, Portugal

* Corresponding author: J. C. Cameselle, Departamento de Bioquímica y Biología Molecular y Genética, Facultad de Medicina, Universidad de Extremadura, Av. Elvas s/n, 06006 Badajoz, Spain; telephone +34-924289470; e-mail: camselle@unex.es

[3] Current address: Clínica Docente los Jarales, Av. El Parque c/c Arterial 31, San Diego 2006, Estado Carabobo, Venezuela

Table S1. Kinetic parameters of the C253V mutant of ADPRibase-Mn. For comparison, similar data of the wild type and the C253A mutant are shown [a]. The results are compatible with the hypothesis that Cys^{253} is a steric constraint that hinders the correct positioning of cADPR in the ADPRibase-Mn active site. The data are means ± S.D. of three experiments. Fold changes (↑, increase; ↓, decrease; ≈, negligible [a]) of the kinetic parameters of the C253V and C253A mutants were calculated with respect to the wild type.

Substrate		C253V	Fold change versus wt	Wild-type[a]	Fold change versus wt	C253A[a]
ADP-ribose	k_{cat} (s^{-1})	91 ± 12	(↑2.6)	35 ± 11	(↑2.8)	97 ± 8
	K_M (μM)	132 ± 12	(↑2.2)	60 ± 8	(↑1.6)	94 ± 6
	k_{cat}/K_M ($M^{-1}s^{-1}$)	690,000 ± 105,000	(≈)	590,000 ± 160,000	(↑1.7)	1,000,000 ± 100,000
CDP-choline	k_{cat} (s^{-1})	109 ± 9	(↑2.2)	50 ± 4	(↑1.6)	79 ± 7
	K_M (μM)	1,120 ± 115	(↑3.2)	350 ± 60	(≈)	500 ± 70
	k_{cat}/K_M ($M^{-1}s^{-1}$)	98,000 ± 2,500	(↓1.5)	150,000 ± 30,000	(≈)	160,000 ± 20,000
2′,3′-cAMP	k_{cat} (s^{-1})	148 ± 8	(↑2.5)	60 ± 13	(≈)	83 ± 5
	K_M (μM)	2,380 ± 250	(≈)	2,400 ± 300	(≈)	2,600 ± 250
	k_{cat}/K_M ($M^{-1}s^{-1}$)	62,300 ± 3,500	(↑2.5)	25,000 ± 6,000	(≈)	32,000 ± 2,000
cADPR	k_{cat} (s^{-1})	1.6 ± 0.1	(↓2.0)	3.2 ± 0.2	(↑2.8)	8.9 ± 0.8
	K_M (μM)	560 ± 180	(≈)	780 ± 200	(↓3.9)	200 ± 40
	k_{cat}/K_M ($M^{-1}s^{-1}$)	3,000 ± 700	(≈)	4,000 ± 1,000	(↑11.0)	44,000 ± 9,000

[a] Data for wild-type ADPRibase-Mn and its C253A mutant are taken from previous work, as is the criterion (increase or decrease lesser than 1.5 fold) for considering change as negligible (reference 55 in the main manuscript).

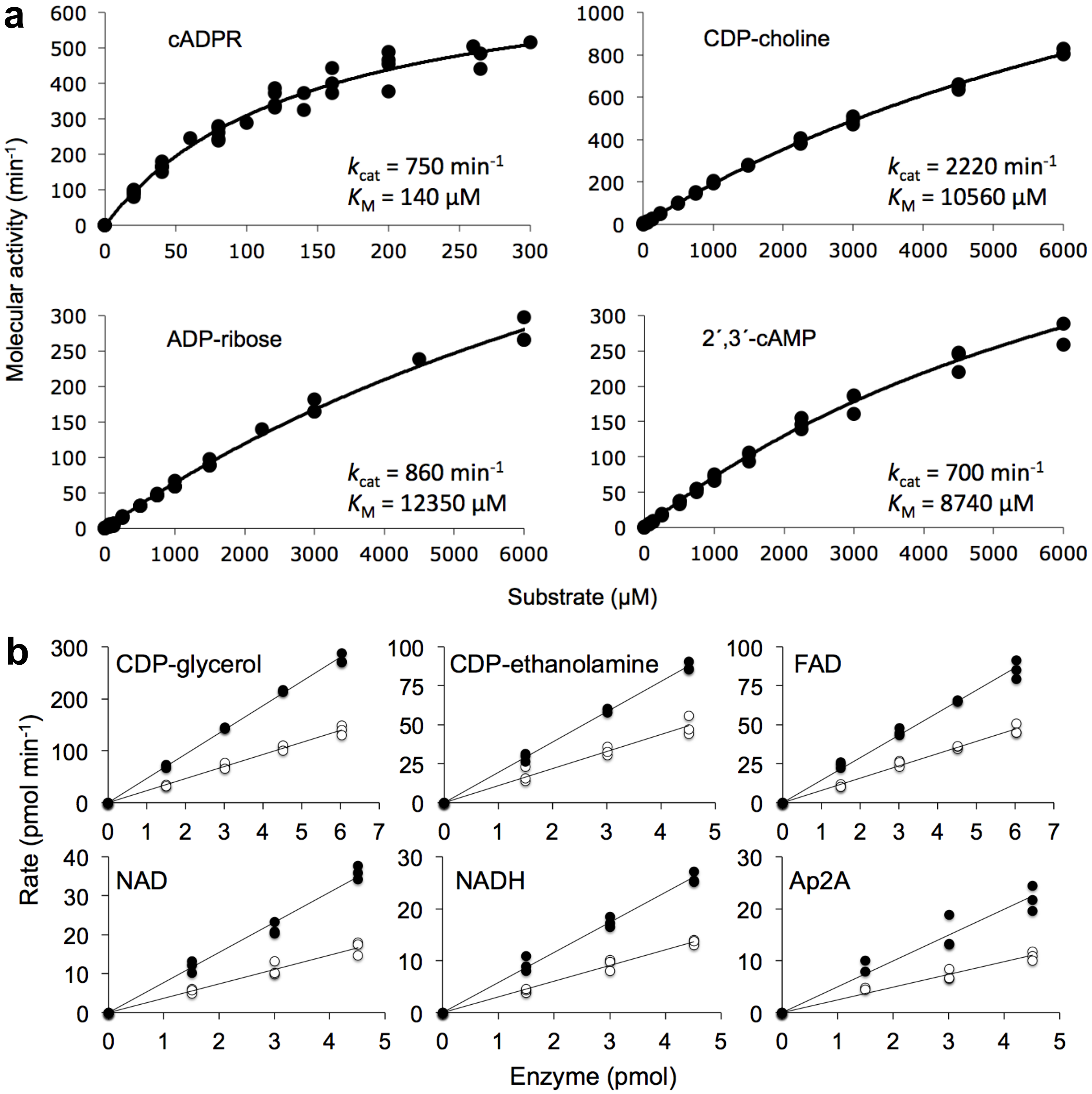

Figure S1. Estimation of the specificity constants or catalytic efficiencies (k_{cat}/K_M) of the cADPR phosphohydrolase obtained by mutagenic engineering of human ADPRibase-Mn. The data correspond to the quadruple mutant F37A+L196F+V252A+C253G-ADPRibase-Mn acting on different substrates. Similar experiments were performed with other mutant proteins. (**a**) Saturation curves that allow separate estimations of k_{cat} and K_M (given in each panel). Data points are reaction rates (molecular activities) obtained with different substrate concentrations in three independent experiments which are pooled in each plot for adjustment. The Michaelis-Menten equation was adjusted by nonlinear regression to each data set using the Solver tool of Microsoft Excel 2011 for the Mac, version 14.1.0. (**b**) Initial-rate assays with different amounts of enzyme at (○) 0.1 mM or (●) 0.2 mM substrate, when reaction rate was (near) proportional to substrate concentration and k_{cat}/K_M = v/([E][S]), [E] being the total enzyme concentration and [S] the concentration of substrate.

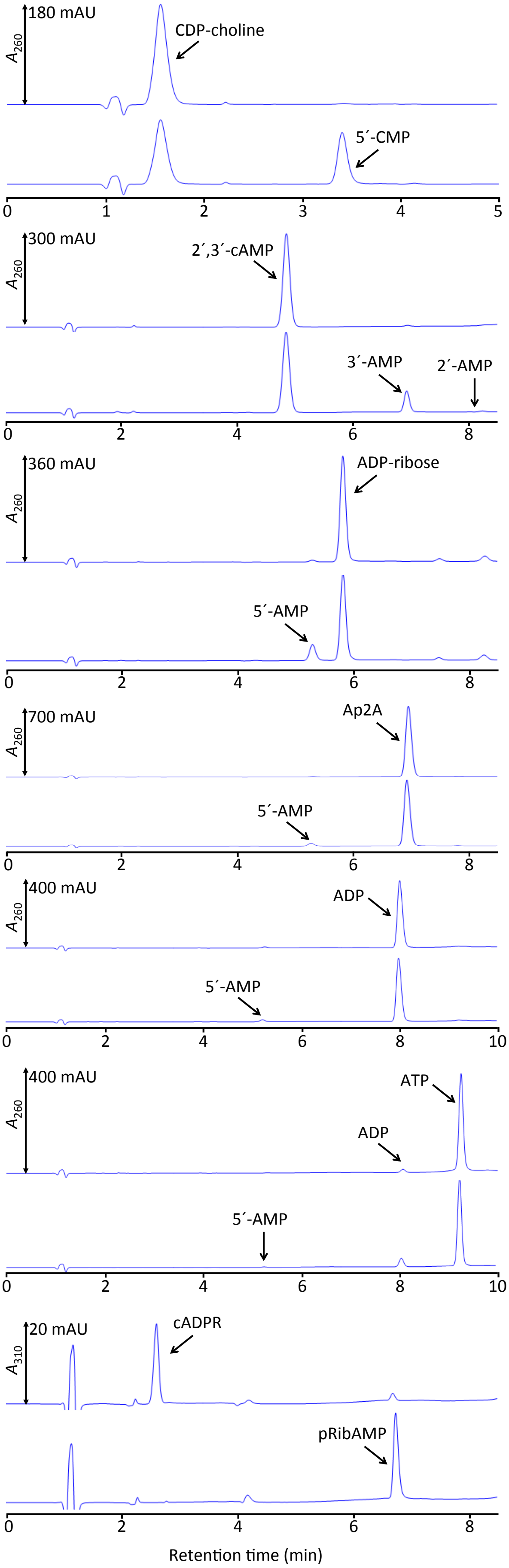

Figure S2. Demonstration of the activity of cADPR phosphohydrolase on individual nucleotides. CDP-choline, 2′,3′-cAMP, ADP-ribose, Ap2A, ADP, ATP or cADPR, each at 0.1 mM concentration were incubated at 37ºC with 4.2 µg ml^{-1} of the specific cADPR phosphohydrolase under otherwise standard conditions. The formation of products was monitored by HPLC at 260 nm (310 nm in the case of cADPR). In each case, the upper chromatogram corresponds to the initial state of the reaction mixture, and the lower one to a 30-min incubation. The identities of the products indicated were confirmed with samples of commercial compounds, or in the case of pRibAMP according to our previous work (ref. 51 in the main text).

The separations were performed in a 200 mm × 2.1 mm octadecylsilica column (Hypersil ODS; Agilent) with a 20 mm × 2.1 mm guard column of the same material. The chromatographic runs were developed at 0.5 ml min^{-1} with a 2-min isocratic elution in 5 mM phosphate, followed by a 4-min linear gradient up to 43 mM phosphate, a 2-min linear gradient up to 100 mM phosphate, and finished with a 4-min isocratic wash, using sodium phosphate buffers adjusted at pH 7.0, and containing 5 mM tetrabutylammonium bromide and 20% (vol/vol) methanol.

The seven compounds studied here individually are those incubated together in the reaction mixture shown in the main text (see the Results section and Fig. 4). These individual incubations were performed with a 4-fold higher enzyme concentration than that used for Fig. 4. Minor differences of retention time between this Fig. S2 and Fig. 4 (including the inversion of elution order of 2′,3′-cAMP and 5′-AMP) are due to the different chromatographic conditions, particularly a different pH and composition (tetrabutylammonium and methanol concentrations) of the mobile phase.
